# Reduced levels of ALS gene *DCTN1* induce motor defects in *Drosophila*

**DOI:** 10.3389/fnins.2023.1164251

**Published:** 2023-06-09

**Authors:** Rebecca Borg, Paul Herrera, Angie Purkiss, Rebecca Cacciottolo, Ruben J. Cauchi

**Affiliations:** ^1^Centre for Molecular Medicine and Biobanking, Biomedical Sciences Building, University of Malta, Msida, Malta; ^2^Department of Physiology and Biochemistry, Faculty of Medicine and Surgery, University of Malta, Msida, Malta

**Keywords:** *Drosophila*, amyotrophic lateral sclerosis, DCTN1, DCTN1-p150, DRED, CG9026, dynactin, CG9279

## Abstract

Amyotrophic lateral sclerosis (ALS) is a rapidly progressive neuromuscular disease that has a strong genetic component. Deleterious variants in the *DCTN1* gene are known to be a cause of ALS in diverse populations. *DCTN1* encodes the p150 subunit of the molecular motor dynactin which is a key player in the bidirectional transport of cargos within cells. Whether *DCTN1* mutations lead to the disease through either a gain or loss of function mechanism remains unresolved. Moreover, the contribution of non-neuronal cell types, especially muscle tissue, to ALS phenotypes in *DCTN1* carriers is unknown. Here we show that gene silencing of *Dctn1*, the *Drosophila* main orthologue of *DCTN1*, either in neurons or muscles is sufficient to cause climbing and flight defects in adult flies. We also identify Dred, a protein with high homology to *Drosophila* Dctn1 and human DCTN1, that on loss of function also leads to motoric impairments. A global reduction of Dctn1 induced a significant reduction in the mobility of larvae and neuromuscular junction (NMJ) deficits prior to death at the pupal stage. RNA-seq and transcriptome profiling revealed splicing alterations in genes required for synapse organisation and function, which may explain the observed motor dysfunction and synaptic defects downstream of *Dctn1* ablation. Our findings support the possibility that loss of *DCTN1* function can lead to ALS and underscore an important requirement for DCTN1 in muscle in addition to neurons.

## 1. Introduction

Amyotrophic lateral sclerosis (ALS) is a rapidly progressing, fatal neurodegenerative disease. Patients mostly present with weakness in either the limb or bulbar muscles resulting from degeneration of upper and/or lower motor neurons. This leads to a gradual decline in their mobility with death typically occurring around 3 years following clinical onset, mostly due to respiratory failure (Brown and Al-Chalabi, 2017; Van Es et al., 2017). Genetics plays a strong role in ALS pathoaethiology, hence, to date, more than 40 genes have been associated with the disease (Goutman et al., 2022). In admixed populations, a sizable number of ALS cases can be explained by dominant causal variants residing in *C9orf72*, *SOD1*, *TARDBP* and *FUS* genes (Zou et al., 2017). Deleterious variants in several other genes including the *DCTN1* gene are a rare cause of ALS globally although a founder effect can inflate their contribution to ALS in homogeneous populations (Borg et al., 2021; Farrugia Wismayer et al., 2023).

The *DCTN1* gene encoding the p150 subunit of the molecular motor dynactin has been identified as an ALS gene nearly two decades ago (Puls et al., 2003; Munch et al., 2004). The discovery underscored impaired axonal transport as a mechanism for motor neuron degeneration in ALS. Dynactin is a key player in the bidirectional transport of cargos including vesicles, organelles, RNAs and proteins along microtubules mediated through either dynein or kinesin (Deacon et al., 2003; Schroer, 2004; Ross et al., 2006; Haghnia et al., 2007). In mice, dominant missense mutations in the *DCTN1* homologue lead to a late-onset, slowly progressive motor neuron disease characterised by gait abnormalities, motor neuron loss, neuromuscular junction (NMJ) defects and, eventually, paralysis (Laird et al., 2008). Heterozygous mutations in *Dctn1* (also referred as *DCTN1-p150*), the homologous gene in *Drosophila* also leads to age-dependent motor deficits, reduced survival and synaptic abnormalities at the NMJ (Eaton et al., 2002; Lloyd et al., 2012). It is thought that missense mutations in the *DCTN1* gene lead to ALS phenotypes through a gain of function mechanism. However, it remains as yet unclear whether haploinsufficiency of DCTN1 plays a role in the disease process. Furthermore, the contribution of non-neuronal cell types, specifically muscle tissue, to ALS phenotypes in patients harbouring *DCTN1* mutations is not known.

Here, we present data demonstrating that reduced levels of Dctn1 specifically in neurons leads to an age-progressive decline in motoric ability and survival in *Drosophila*. Muscle-selective knockdown leads to similar phenotypes that nonetheless manifest in younger adult flies. We further identify a gene, named here as *Dctn1-related* or *Dred*, as having homology to *Drosophila Dctn1* and human *DCTN1*. Loss of *Dred* function in either muscle or neurons also leads to motor impairments. Interestingly, a global reduction of Dctn1 leads to paralysis of larvae and NMJ defects. Finally, RNA sequencing (RNA-seq) of these organisms followed by transcriptome analysis led to the identification of alterations in genes required for synapse organisation and function, hence allowing us to speculate that these may explain the motor dysfunction and synaptic defects downstream of *Dctn1* deficiency.

## 2. Methods

### 2.1. Fly culture and stocks

Flies were cultured on food consisting of sugar, corn meal, yeast and agar in plastic vials at an incubation temperature of 25°C under 12 h day/night cycles. The RNAi transgenic constructs *Dctn1-IR^1^* (ID: 3785), *Dred-IR^1^* (ID: 105109) and *Dred-IR^2^* (ID: 45052) were obtained from the Vienna *Drosophila* Resource Centre, Austria (Dietzl et al., 2007). The *Dcr-2* transgene and the GAL4 drivers were obtained from the Bloomington *Drosophila* Stock Centre (NIH P40OD018537) at Indiana University, United States. Constitutive expression of transgenes was driven by the *Act5C*-GAL4 whereas the *Mef2*-GAL4 and *elav*-GAL4 drivers were employed to induce expression specific to muscle and neurons, respectively. Combination of the various genetic tools was performed according to standard genetic crossing schemes.

### 2.2. Protein alignment

To determine % amino acid similarity and identity between human DCTN1 (NP_004073.2) and its *Drosophila* orthologues Dctn1 (NP_524061.1) and Dred (NP_649124.1), we utilised the DRSC Integrative Ortholog Prediction Tool (DIOPT, https://www.flyrnai.org/diopt). Alignment of the *Drosophila* proteins with their human counterpart was performed by Clustal Omega (EMBL-EBI).

### 2.3. Neuromuscular function assays

Larval mobility was assessed at 72 h (L3a) and 96 h (L3b) after egg laying. Briefly, third instar (L3) larvae (sex ratio, 1:1) of the appropriate genotype were first place on a 0.7% agar plate and allowed to acclimatise for 5 min. Subsequently, the number of forward body wall contractions exhibited by the organism in 30 s were counted. Each larva was assessed three times before an average was taken. A minimum of 15 larvae per genotype were assayed.

Climbing performance of male adult flies was assessed at different timepoints following eclosion. In brief, two empty polystyrene tubes were vertically joined by tape facing each other. Flies (15–20) were then transferred to the lower tube and allowed to acclimatise. Flies were then gently tapped down to the bottom of the tube. To determine the percentage climbing success rate, the number of flies per group, that climb above the 8 cm mark by 10 s were counted. For determination of the time for first fly, the time taken for the first fly within a group to cross the 8 cm mark was observed. Four trials were performed for each group of flies and a minimum of four groups were assayed per genotype.

Assessment of flight performance was determined on male adult flies through the use of the Droso-Drome apparatus as described previously (Lanfranco et al., 2017). This consisted of a 1 L glass bottle coated with an alcohol-based sticky fluid, and divided into 4 sectors, of 5 cm each, spanning a total height of 20 cm. In short, flies first underwent a ‘warm-up’ by inducing negative geotaxis in an empty tube for 3 times. Organisms were then dropped into the Droso-Drome to induce flight. The number of flies distributed in each sector was next counted, divided by the total number of flies dropped and multiplied by 100 to generate the percentage number of flies per sector. Fight ability correlates with the sector in which flies are distributed on landing, hence, fly percentages that are skewed towards the lower sectors of the Droso-Drome are indicative of reduced flight capacity. Four trials were performed for each group of flies and a minimum of four groups were assayed per genotype.

### 2.4. Assessment of adult fly survival

Male adult flies were maintained in vials at a density of 15 to 20 flies per vial. The percentage number of flies alive at each time point measured was determined by dividing the number of flies still alive by the initial number of flies in the vial and multiplying the value by 100. During their adult lifespan, flies were transferred to new vials routinely.

### 2.5. Immunohistochemistry of NMJs

Wandering L3 larvae were dissected in phosphate buffered saline (PBS) to expose the body wall muscles, then fixed in 4% paraformaldehyde in PBS and washed in PBS + 0.1% Triton X-100 (PBT). Tissues were then stained overnight at room temperature by mouse anti-Discs large antibody (1:1,000; Developmental Studies Hybridoma Bank, University of Iowa, United States). On the following day, tissues were washed in PBT and stained overnight at room temperature with anti-mouse Alexa Fluor 488-conjugated secondary goat antibody (1:50). After a final wash in PBT, the samples were mounted in 90% glycerol with anti-fade. Imaging was performed with the Optika B-600TiFL microscope (20x or 40x objectives) using brightfield and fluorescent light channels.

### 2.6. Analysis of NMJ morphology parameters

Analysis of NMJ morphology was done as described previously (Cacciottolo et al., 2019). The area of NMJs innervating ventral longitudinal muscles 6 and 7 derived from abdominal segments 2–3 was quantified by the ImageJ software (NIH). Branch number was determined by counting the number of arborisations containing at least two boutons within a single NMJ. To determine bouton numbers, all boutons were counted within a single NMJ.

### 2.7. RNA extraction

RNA was extracted from 12–15 L3b larvae of the desired genotype using the Qiagen RNeasy Plus Mini Kit (Qiagen, Hilden, Germany) following manufacturer’s instructions. For quantitative RT-PCR, sex ratio of larvae was 1:1 whereas for RNA-seq, RNA was extracted from females larvae only. In brief, whole larvae were homogenized and lysed. Tissue lysates were then spun through genomic DNA eliminator spin columns to remove genomic DNA and RNeasy Mini spin columns were subsequently used to purify total RNA.

### 2.8. Quantitative RT-PCR

Quantification of *Dctn1* and *Dred* expression levels was achieved by amplifying the corresponding cDNA using the SOLIScript 1-step SolisGreen kit (Solis Biodyne, Tartu, Estonia) following manufacturer’s instructions. The primers were from Integrated DNA Technologies (Leuven, Belgium) and were specific for *Dctn1* (forward: 5′ – CGCACCAAGGAGAAGCTTAG – 3′; reverse: 5′ – GGTCGCGATCATAGATGGTT – 3′), *Dred* (forward: 5′ – CACGGCAGCATTTACTTCAA – 3′; reverse: 5′ – GAGTCGCCAAAAATTTTCCA – 3′) and housekeeping gene *RpL32* (forward: 5′ – TACAGGCCCAAGATCGTGAA – 3′; reverse: 5′ – GACAATCTCCTTGCGCTTCT – 3′). The transcriptional levels were calculated by the 2–ΔΔCt (Ct, cycle of threshold) method. ΔΔCt = ΔCt of experimental group – mean ΔCt of control groups. ΔCt = Ct (gene of interest) − Ct (housekeeping).

### 2.9. RNA-seq and data analysis

RNA-seq libraries from RNA samples (derived from female L3 larvae) were prepared and sequenced at the Beijing Genomics Institute, Denmark as described previously (Borg et al., 2023). Briefly, poly(A) mRNA was enriched using poly(T) oligo-attached magnetic beads. This was followed by fragmentation and subsequent first strand cDNA synthesis using random hexamer N6 primers and reverse transcriptase. Following end repair and adaptor ligation, cDNA fragments were PCR amplified and purified to generate single-stranded DNA circles in a final library. DNA nanoballs were finally generated by rolling circle replication, which underwent paired end sequencing (100 bp) on the BGI DNBseq platform.

Raw reads were filtered using SOAPnuke (Li et al., 2009) and clean reads were mapped to the reference *Drosophila* genome using HISAT2 (Kim et al., 2019). Transcript quantification was obtained using RSEM and normalized as fragments per kilobase of transcript per million mapped reads (FPKM) (Li and Dewey, 2011). Differentially expressed genes (DEGs) were identified by the DESeq2 algorithm with *p*-values adjusted for multiple comparisons by the Benjamini and Hochberg procedure, and differential expression of the genes determined using a false discovery rate (FDR) cut off of <0.05 (Love et al., 2014). DEGs with a > 2 fold change (log2FC > 1) were selected. Differentially spliced genes (DSGs) were detected using rMATS (Shen et al., 2014) and five types of alternative splicing events including skipped exon (SE), alternative 5′ splicing site (A5SS), alternative 3′ splicing site (A3SS), mutually exclusive exons (MXE) and retained Intron (RI) were defined. GO biological pathway analysis on DSGs and upregulated or downregulated DEGs was carried out using ShinyGO (Ge et al., 2020).

### 2.10. Statistical analysis

Values are presented as means ± SEM unless otherwise indicated. The unpaired *t*-test was used to compare measures between 2 groups whereas two-way ANOVA, followed by Dunnett’s *post hoc* test, was used for multiple comparisons with control (GraphPad Prism v9.4.1). Differences were deemed statistically significant if *p* < 0.05.

## 3. Results

### 3.1. Knockdown of the Drosophila DCTN1 orthologues *Dctn1* and *Dred*

The top-most predicted orthologue of DCTN1 in *Drosophila* is Dctn1 (CG9206). Compared to its human counterpart, Dctn1 has an amino acid similarity and identity of 54 and 33%, respectively (93% coverage) (Supplementary Figure S1A). Nonetheless, within the *Drosophila* genome we were able to identify a second gene (*CG9279*), named here as *Dctn1-related* or *Dred,* that encodes a protein that also has a high homology to DCTN1. Hence, Dred is 41% similar and 25% identical (74% coverage) to human DCTN1 (Supplementary Figure S1B). Dred and Dctn1, which compared to each other have 48% amino acid similarity and 31% amino acid identity (77% coverage), can be considered as paralogues. It must also be noted that the locus of *Dred* is shared with another gene (*CG46434*).

**Figure 1:**
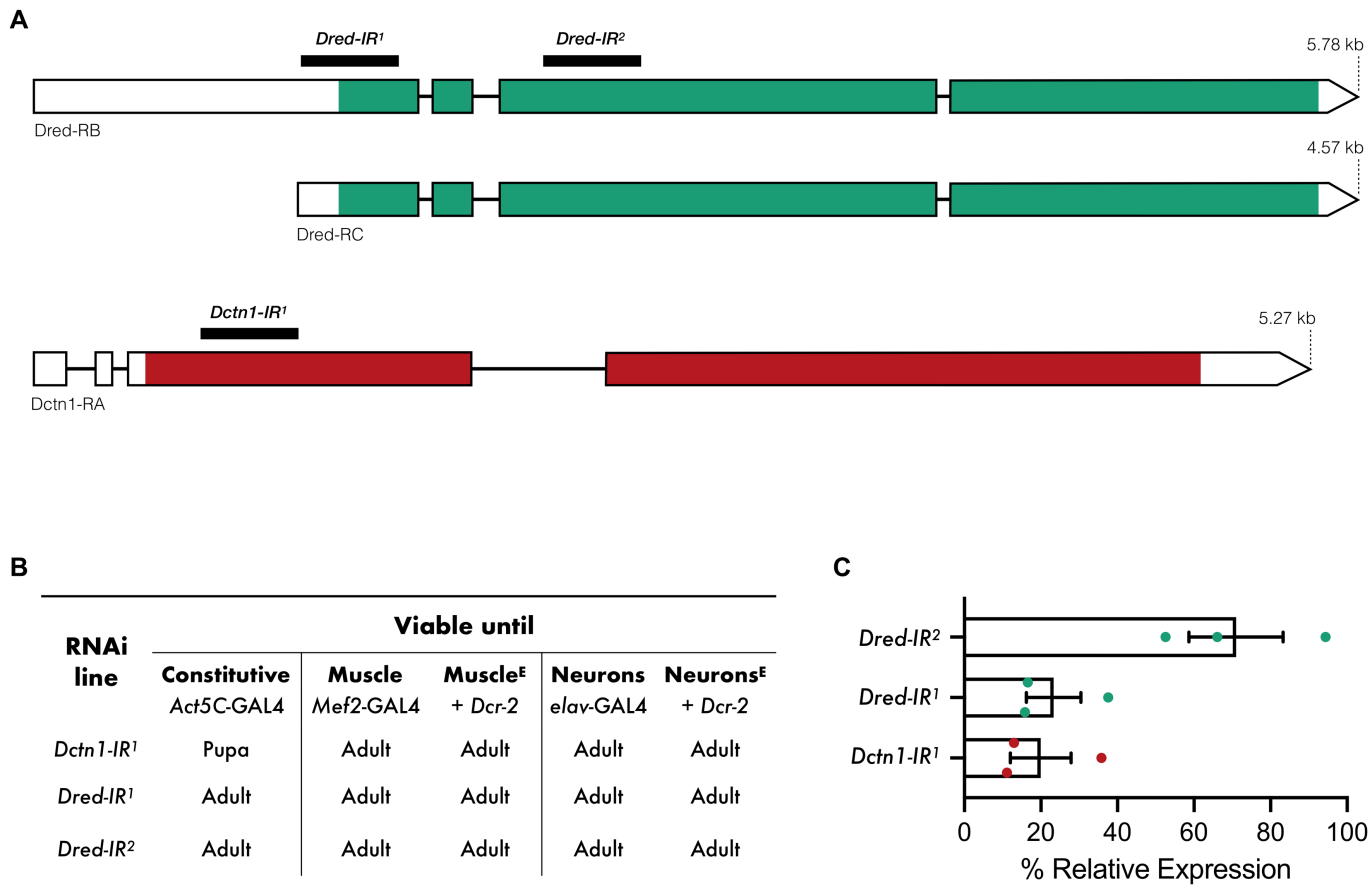
Viability outcomes and transcript expression on activation of RNAi transgenes targeting *Dctn1* or *Dred*. **(A)** Predicted *Dred* and *Dctn1* mRNA transcripts in *Drosophila* and regions targeted by RNAi constructs. **(B)** Viability outcomes of flies in which each RNAi transgene was activated either constitutively or in specific tissues by the indicated GAL4 drivers. Co-expression of Dcr-2, as indicated by E in superscript, was employed to enhance tissue-specific knockdown. **(C)** Expression of *Dctn1* or *Dred* relative to the housekeeping *RpL32* gene in L3b larvae in which the indicated RNAi transgene was constitutively expressed as determined by qRT-PCR. Each bar represents the mean ± SEM of three biological replicates with the respective data points superimposed on the bars.

We employed the *UAS/*GAL4 system to activate RNAi transgenes targeting either *Dctn1* or *Dred* predicted mRNA transcripts (Figure 1A). On RNAi activation in the whole organism (*Act5c*-GAL4), constitutive knockdown of *Dctn1* was found to halt developmental progression, leading to flies perishing at the pupal stage. The stage of death is therefore later than that observed for null mutations in *Dctn1*, which are embryonic or early larval recessive lethals (Harte and Kankel, 1982). Global silencing of *Dred* had no effect on adult fly viability (Figure 1B). Selective expression of the *Dctn1*-specific RNAi transgene in either neurons (*elav*-Gal4) or muscle (*Mef2*-Gal4), even when knockdown was enhanced by co-expression of the *Dcr-2* transgene, was found to bypass lethality, hence, leading to viable adult flies (Figure 1B).

We next assessed gene knockdown efficiency and specificity by performing quantitative RT-PCR (qRT-PCR) on RNA extracted from third instar (L3) larvae with constitutive expression of each transgenic construct. We show that activation of the *Dctn1-IR^1^* transgene, which targets the 5′ coding sequence of the *Dctn1* transcript, leads to a strong reduction in *Dctn1* transcript expression (20%) (Figure 1C). A robust knockdown was also observed on constitutive activation of the *Dred-IR^1^* transgene (23%) which targets the 5′ untranslated region and part of the exon 1 of the *Dred* transcript (Figure 1C). Moderate reduction in transcript levels (71%) were however noted for the *Dred-IR^2^* transgene which targets a downstream coding region of the *Dred* transcript (Figure 1C).

### 3.2. Motor impairment in adult flies with loss of *Dctn1* or *Dred* function

**Figure 2:**
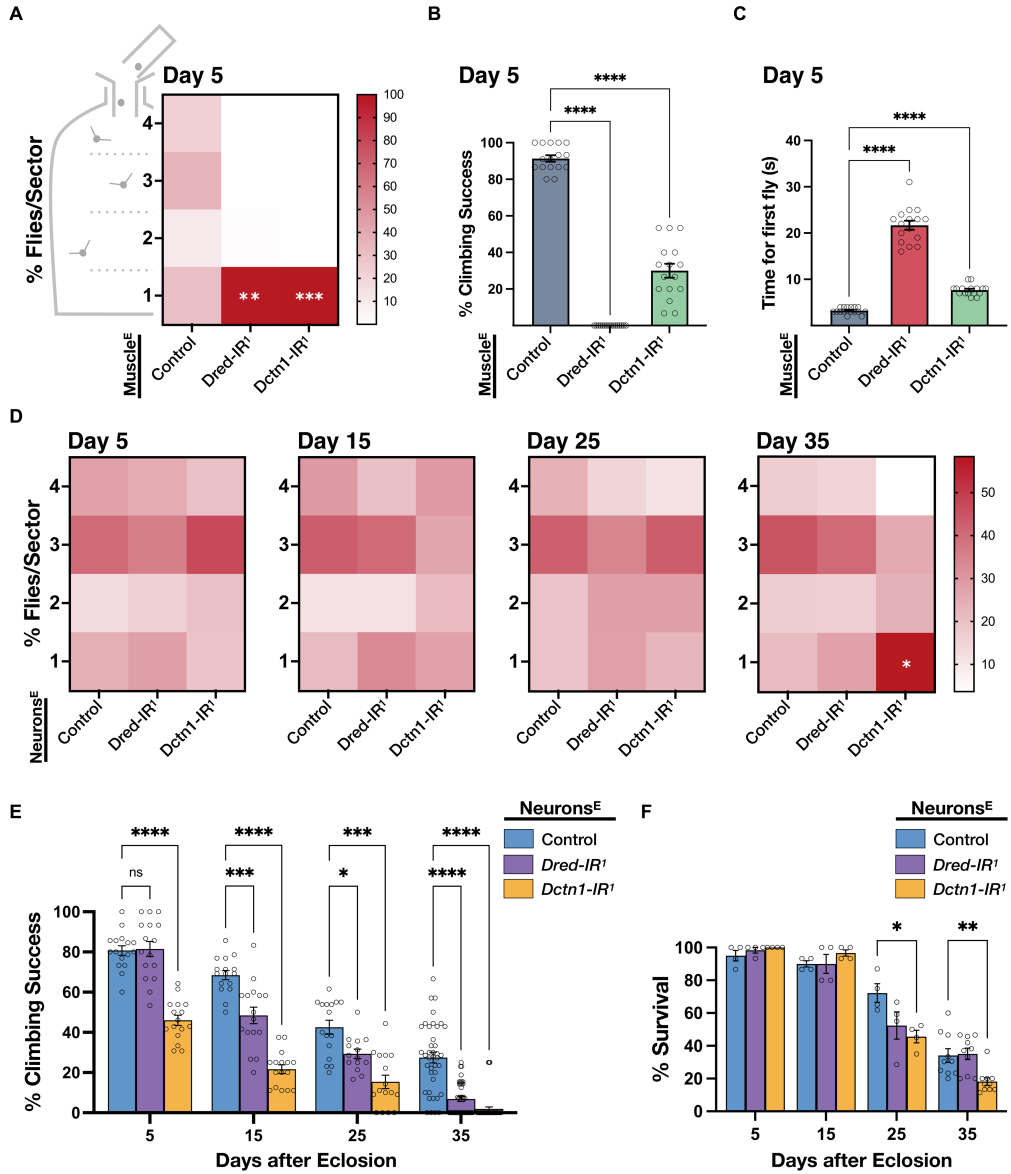
Knockdown of *Dctn1* or *Dred* induces motor deficits. **(A)** Heat map showing percentage distribution of flies landing in either of four sectors (4, top, 1, bottom) of the Droso-Drome apparatus after drop-off (4 replicates/genotype, *n* = 15 flies/replicate). Young adult flies with muscle-selective (*Mef2*-GAL4) expression of the indicated RNAi transgenes, enhanced by *Dcr-2*, had significant flight defects compared to the control. **(B)** Climbing ability of young adult flies with enhanced reduction of Dctn1 or Dred in muscle was significantly impaired compared to the control. **(C)** Time taken for the first fly out of a sample population to reach a predetermined threshold was significantly longer in flies in which *Dred-IR^1^* or *Dctn1-IR^1^* activation was restricted to muscle tissue. **(D)** Heat maps showing percentage number of organisms per sector for flies with neuron-selective (*elav*-GAL4) expression of the indicated RNAi transgenes, enhanced by *Dcr-2*, that were assessed at different timepoints throughout adulthood and compared to age-matched controls (≥4 replicates/genotype, *n* ≥ 15 flies/replicate). Flight defects were apparent on brain-specific knockdown of *Dctn1* in old flies. **(E)** Climbing ability of adult flies with enhanced brain-exclusive *Dctn1* or *Dred* loss of function compared to control and assessed at different time points. **(F)** Survival of adult flies in which Dctn1 or Dred RNAi was induced specifically in neurons. Flies with neuron-specific *Dctn1* knockdown have reduced survival during late stages of adulthood. For graphs, each bar represents the mean ± SEM of at least 4 independent experiments superimposed on the bars (for each genotype, *n* ≥ 15 flies/replicate). Significance was tested by two-way ANOVA with Dunnett’s *post hoc* test and for all data, ^*^p = 0.01, ^**^p < 0.01, ^***^p < 0.001, and ^****^p < 0.0001. Enhanced knockdown was achieved by co-expression of *Dcr-2* (indicated by E in superscript). Control expressed both driver and *Dcr-2*.

Given that the *Dctn1-IR^1^* and *Dred-IR^1^* transgenic constructs we identified were sufficient to decrease the respective transcript levels to a high degree, we asked whether loss of function of either *Dctn1* or *Dred* in disease-relevant tissues leads to an impairment in motoric ability, which is considered as the most obvious outward feature of ALS. First, we induced a knockdown of either *Dctn1* or *Dred* in muscle tissue, enhanced by co-expression of Dcr-2. Interestingly, the resulting adult flies had severe flight defects as early as day 5 post-eclosion as observed by a significant percentage that was distributed to the lower most sector (sector 1) of the Droso-Drome apparatus (Figure 2A). Climbing ability was also profoundly reduced in flies with muscle-specific *Dctn1* knockdown and totally abolished in young flies with muscle-exclusive loss of *Dred* function (Figure 2B). To better document the climbing defects we then assessed the time taken for the first fly out of a sample population to reach a predetermined threshold. Compared to control organisms, we observed a 2-fold or 6-fold increase in the time taken for flies with *Dctn1* or *Dred* muscle-selective gene silencing, respectively (Figure 2C). Although not effected in day 5 old flies, survival underwent a drastic reduction at day 15 post-eclosion only in flies with muscle-selective *Dred* knockdown (Supplementary Figure S2). We also note that motoric defects were specific to the adult stage given that we did not detect any locomotor abnormalities in larvae with the respective genotype compared to control (Supplementary Figure S3).

Next we generated adult flies with neuron-specific knockdown of *Dctn1* or *Dred* enhanced by co-expression of Dcr-2. We observed that flies had no significant flight behaviour differences compared to control until late adulthood, where a significant number of flies with brain-selective knockdown of *Dctn1* but not *Dred* were flight impaired which is indicative of an age-dependent decline in flight capacity (Figure 2D). On assessment of climbing ability, we noticed that reduced levels of either Dctn1 or Dred, specifically in neurons, induced an age-progressive decline in the climbing performance success rate, which started earlier and was relatively worse in flies with *Dctn1* knockdown (Figure 2E). Survival was also negatively affected in day 25- and day 35-old adult flies with neuron-selective activation of the *Dctn1-IR^1^* but not *Dred-IR^1^* transgene (Figure 2F). We did not observe any motoric defects in larvae with neuron-selective loss of either *Dred* or *Dctn1* (Supplementary Figure S3). In summary, these findings support an important role for Dctn1 or Dred in normal neuromuscular behaviour of adult flies, required in both compartments of the motor unit.

### 3.3. Constitutive *Dctn1* deficiency induces reduced muscle contraction and NMJ defects in larvae

**Figure 3:**
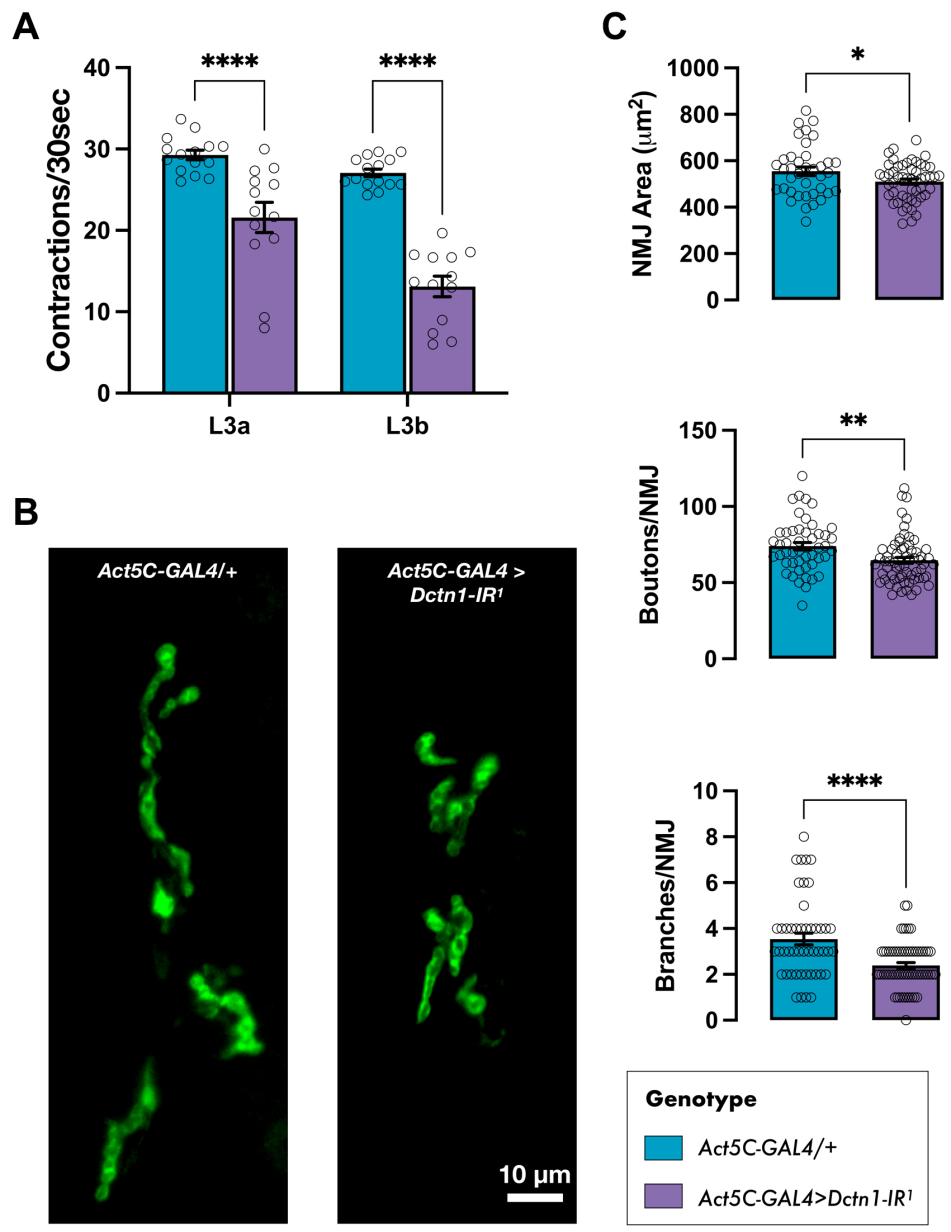
Loss of *Dctn1* disrupts larval mobility and induces NMJ defects. **(A)** Body wall contraction rate of L3 larvae with global downregulation of *Dctn1* assessed at 72 h (L3a) and, subsequently, 96 h (L3b) after egg laying (*n* ≥ 13/genotype). **(B)** Representative DLG-stained NMJs innervating ventral longitudinal muscles 6 and 7 in L3b larvae with constitutive knockdown of *Dctn1*. **(C)** Quantification of various NMJ parameters including synaptic area, bouton numbers and number of branches per NMJ (*n* ≥ 38 NMJs derived from ≥15 larvae/genotype). For graphs, each bar represents the mean ± SEM of several independent experiments superimposed on the bars. Significance was tested by two-way ANOVA with Dunnett’s *post hoc* test or the unpaired *t*-test and for all data, ^*^p = 0.01, ^**^p < 0.01, and ^****^p < 0.0001.

Considering that constitutive knockdown of *Dctn1* induced lethality prior to the adult stage, we wondered whether we could also uncover neuromuscular deficits in larvae during an earlier developmental stage. To this end, we focused on third instar (L3) larvae and assessed their mobility. We show that flies with global *Dctn1* knockdown experienced a significant drop in the body wall contraction rate at the early L3a stage (72 h after egg laying) that declined further after the next 24 h (L3b wandering stage) (Figure 3A). We next questioned whether we can link the motor deficits observed on loss of *Dctn1* function with defects in motor synapses, which are a well-recognised sign of ALS pathophysiology (Dadon-Nachum et al., 2011; Verma et al., 2022). To this end, we dissected wandering L3b larvae and examined the NMJs of motor neurons innervating their abdominal muscles. On visual inspection, we observed that loss of *Dctn1* function induced an obvious decrease in NMJ span and complexity (Figure 3B). To quantify these defects, we measured several NMJ morphology parameters including area, number of branches and bouton numbers which were all significantly depressed upon reduction in Dctn1 levels (Figure 3C). In sum, we present data that underscore a role for Dctn1 in synapse organisation and function within the NMJ.

### 3.4. Transcriptional response to loss of *Dctn1*

**Figure 4:**
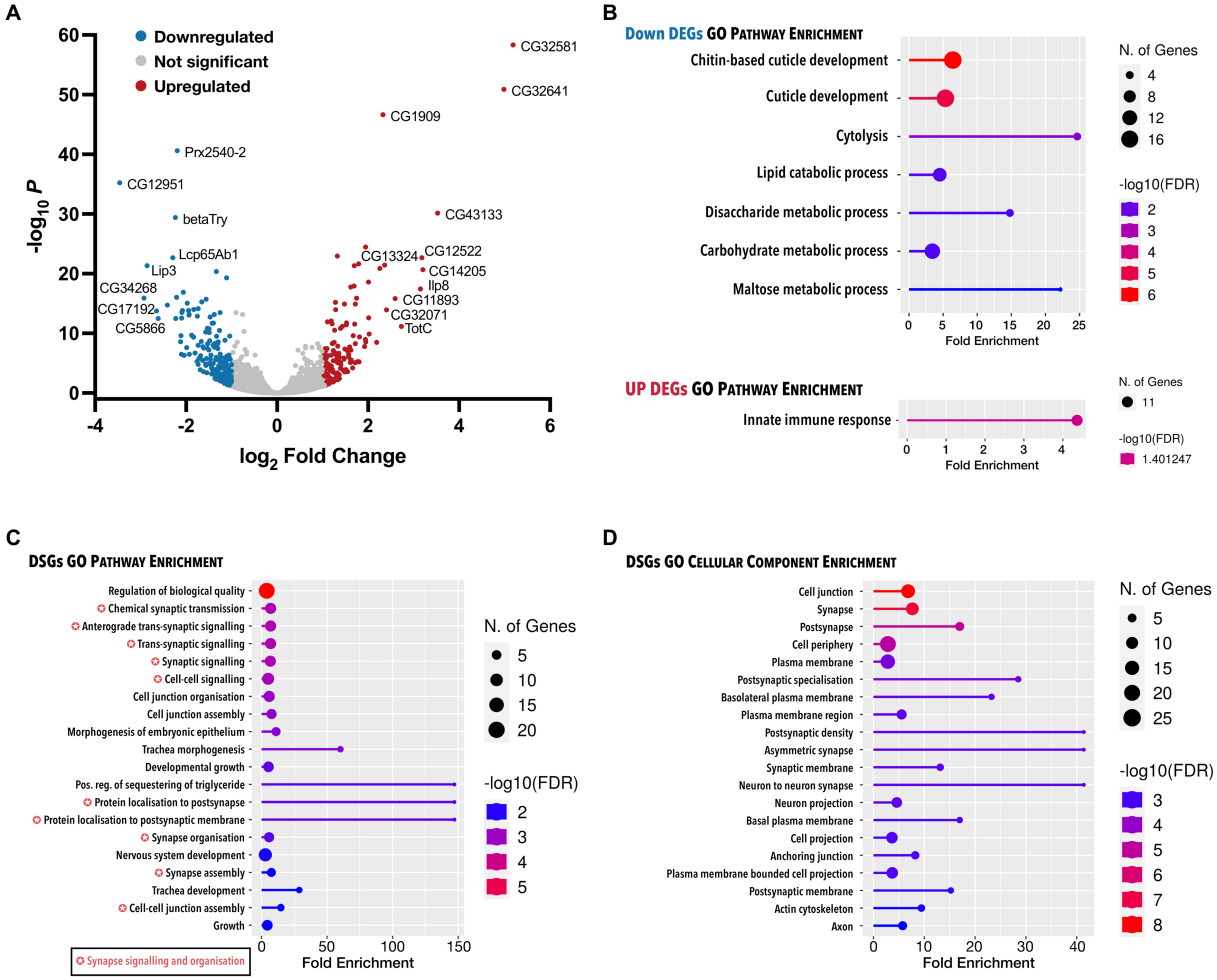
Analysis of all genes with expression and splicing changes in response to *Dctn1* gene silencing. **(A)** Volcano plot showing DEGs in L3b larvae with ubiquitous *Dctn1* knockdown compared to the driver only control (*n* = 3 biological replicates, sex = females). Topmost significant DEGs have been annotated. **(B)** Lollipop plot presenting significant molecular pathway terms enriched in downregulated or upregulated DEGs upon gene ontology (GO) analysis. **(C)** Lollipop plot showing the most significant GO molecular pathways terms enriched in DSGs. **(D)** Lollipop plot exhibiting the most significant GO cellular component terms enriched in DSGs. In **B–D**, GO terms are sorted by FDR (<0.05) with the colour of the lollipops representing the values of the enrichment analysis relative to the other displayed terms (brighter red is more significant) and the size of the dots represent the number of genes that consist the term. GO terms tagged with a colour-coded star indicate pathway overlap.

Finally, to identify the molecular changes responsible for the neuromuscular deficits downstream of *Dctn1* loss of function, we carried out RNA-seq in larvae with constitutive downregulation of *Dctn1*. We found 317 differentially expressed genes (DEGs) of which 176 were downregulated and 141 were upregulated (Figure 4A, Supplementary Material Dataset S1). Only 2 downregulated transcripts were annotated as novel. Gene Ontology (GO) biological pathway enrichment analysis on DEGs revealed a downregulation of processes associated with cuticle development, cytolysis and metabolism, and an upregulation in the innate immune response (Figure 4B). Interestingly, RNA-seq also revealed 59 differentially spliced genes (DSGs) upon *Dctn1* gene silencing, of which 10 had an alternative 3′ splice site (A3SS), 17 had an alternative 5′ splice site (A5SS), 10 had a mutually exclusive exon (MXE), 23 had a retained intron (RI) and 14 had a skipped exon (SE) (Supplementary Material Dataset S2). Several transcripts were subjected to more than one mode of alternative splicing, and the transcript encoded by the polyubiquitin gene *Ubi-p63E* (*CG11624*) was affected by all modes (Supplementary Figure S4). GO biological pathway analysis on DSGs revealed an enrichment of terms associated with synapse signalling and organisation including protein localisation (Figure 4C, Supplementary Material Dataset S3). The synapse was also one of the most enriched GO cellular component terms (Figure 4D). Overall, RNA-seq data revealed several transcriptome alterations that may explain the synaptic deficits and the consequential motor dysfunction resulting from loss of *Dctn1* function.

## 4. Discussion

Mutations in *DCTN1* have been detected in ALS patients of diverse ancestries (Puls et al., 2003; Munch et al., 2004, 2005; Liu et al., 2017; Ryan et al., 2019; Wei et al., 2019; Borg et al., 2021; Farrugia Wismayer et al., 2023). It is well known that the protein encoded by *DCTN1* plays a crucial role in the bidirectional transport of cargos along microtubules in axons of motor neurons (Schroer, 2004). Studies in mice have shown that disruption of *DCTN1* induces ALS-like phenotypes which are accompanied by defects in vesicular transport including excessive synaptic vesicle protein accumulation at NMJs (Lai et al., 2007; Laird et al., 2008). However, it remains unresolved whether loss as opposed to gain of DCTN1 function is a contributing factor to the disease process. Moreover, the contribution of cell types other than neurons to the phenotype in ALS patients carrying *DCTN1* mutations is still unclear. Here, we attempted to address these questions by inducing RNAi-mediated gene silencing of two putative orthologues of DCTN1 in flies. Our findings demonstrate that, indeed, loss of *Dctn1* or *Dred* function is sufficient to induce ALS-like phenotypes in *Drosophila*. Interestingly, we find that in addition to neurons, disruption of DCTN1 orthologues in muscle tissue also impaired motoric ability. *Dctn1* deficiency was also found to induce NMJ defects that overlapped with those observed in animal models carrying *DCTN1* mutations (Eaton et al., 2002; Chevalier-Larsen et al., 2008; Laird et al., 2008; Lloyd et al., 2012). Splicing alterations in genes with a function in synapse organisation and function identified through transcriptome profiling may explain the motor dysfunction and synaptic defects observed in flies with loss of *Dctn1* function.

Considering that missense mutations in *DCTN1* have been associated with ALS under a dominant disease model, a gain of function mechanism has predominated the view of how disease arises in patient carriers. This is supported by reports showing that ALS-linked mutations in *DCTN1* disrupt the folding of its encoded protein to induce aggregates that are toxic to motor neurons (Levy et al., 2006; Laird et al., 2008). Nonetheless, there is evidence that *DCTN1* mutations can disrupt the binding between dynactin and microtubules, which can lead to impaired dynein/dynactin-based transport along microtubules (Levy et al., 2006). *DCTN1* was also found to be downregulated in spinal motor neurons isolated from autopsied patients with sporadic ALS (Jiang et al., 2005). These findings raise the possibility that a loss of function mechanism can also lead to the disease, at least in combination with a toxic gain of function. Here, we show that loss of *Dctn1* function in flies is alone sufficient to induce ALS-like phenotypes and pathology including impaired motoric ability and NMJ defects. Our study is therefore supportive of the possibility that haploinsufficiency arising from *DCTN1* mutations can lead to ALS. Our findings are corroborated by reports demonstrating severe motoric behavioural defects and/or NMJ instability, similar to those described here, upon depletion of the DCTN1 homologues in *C. elegans* (Ikenaka et al., 2013), zebrafish (Bercier et al., 2019) or mouse (Yu et al., 2018). We must however note that the phenotypes we have observed here are resulting from reductions in *Dctn1* levels well below the 50% reduction that is expected in ALS patients harbouring damaging variants in one copy of the *DCTN1* gene. Nonetheless, expression levels of ubiquitous or housekeeping proteins vary between cell types (Groen et al., 2018) so it is plausible that motor neurons or muscle might have lower DCTN1 expression and haploinsufficiency can therefore lead to negative consequences that are greater in these tissues.

Missplicing of several genes involved in synapse organisation and function may explain the motor behavioural phenotypes and NMJ defects we observed in flies with loss of *Dctn1* function. However, it is still unclear how reduced levels of a protein involved in intracellular transport leads to such consequential changes. There is emerging evidence that pre-mRNA splicing can occur outside the nucleus including in axons where spliceosome components and splicing factors, that retain their potential to promote pre-mRNA splicing, have been localised (Glanzer et al., 2005; Giorgi et al., 2007; Konig et al., 2007; Racca et al., 2010; Cosker et al., 2016; Thomas-Jinu et al., 2017; Poulopoulos et al., 2019). To this end, SNRNP70, a component of the major spliceosome, was recently found localised in RNA-associated granules in axons of zebrafish motor neurons and its extra-nuclear requirement for alternative pre-mRNA splicing was found to be important for neuromuscular synaptogenesis (Nikolaou et al., 2022). This also ties well with the identification of intron-retaining transcripts in the dendrites of mature neurons (Giorgi et al., 2007; Ortiz et al., 2017; Sharangdhar et al., 2017). It is therefore plausible that *Dctn1* deficiency can impair anterograde transport of splicing regulators reducing their availability within the cytoplasmic pool with this having a negative impact on the correct RNA processing of proteins required for assembly and operation of the NMJ.

Our work also highlights an important requirement for DCTN1 in muscle tissue in addition to neurons. Data showing rapid motor behavioural abnormalities in young flies with muscle-selective knockdown indicates that muscle appears to be more vulnerable to reduced levels of Dctn1 or Dred. The contribution of muscle tissue to ALS pathophysiology is supported by various studies in animal models (Loeffler et al., 2016; Anakor et al., 2022) including *Drosophila*, where for instance, motor abnormalities that overlap with those described here were reported on muscle-selective disruption of TDP-43, which is itself found aggregated in the majority of ALS patients (Diaper et al., 2013). Whether ALS originates in skeletal muscle leading to motor neuron death through a retrograde signalling cascade, the so-called ‘dying-back’ hypothesis, remains controversial and highly debatable. However, it is highly likely that muscle provides trophic support to motor neurons (Kablar and Belliveau, 2005) and, these signals may be absent either because they fail to be retrogradely transported from the periphery to the cell bodies in motor neurons or because they are not being produced by the muscle itself. The latter hypothesis might be especially true for patients with *DCTN1* mutations. In support, muscle-targeted knockout of BICD2, the causative factor of Spinal Muscular Atrophy Lower Extremity Predominant (SMALED), a lower motor neuron disease, was found to be a major driver of the motor neuron loss in a mouse model (Rossor et al., 2020). BICD2 is a cargo adaptor protein, which binds to the dynein/dynactin transport complex.

In conclusion, our evidence is supportive of the possibility that loss of *DCTN1* function is a likely contributor to ALS with changes in muscle in addition to neurons driving motor system dysfunction. Future work needs to address the tissue-specific contributions to the NMJ deficits observed on ubiquitous *Dctn1* knockdown, focusing on pre-synaptic terminals in addition to post-synaptic NMJ structures. We find it plausible to speculate that impaired splicing of genes required for motor synapse assembly, structure and function lead to the motor dysfunction phenotypes as well as the evident NMJ defects downstream of *Dctn1* loss of function. In addition to future work directed at confirming this link, we therefore anticipate that our study will be a stimulus for further investigations into the mechanisms through which loss of *DCTN1* triggers neuromuscular-specific splicing changes.

## Data availability statement

The datasets presented in this study can be found in online repositories. The names of the repository/repositories and accession number(s) can be found at: https://www.ncbi.nlm.nih.gov/geo/, GSE225648.

## Author contributions

RuC conceptualized and designed the experiments. RB, AP, RC, PH, and RuC performed experiments. RuC, RB, and AP analysed and interpreted the data. RuC wrote the manuscript. All authors contributed to the article and approved the submitted version.

## Funding

This work was supported by the Malta Council for Science & Technology Fusion R&I Research Excellence Programme, the University of Malta Research Seed Fund, the Malta Council for Science & Technology Internationalisation Partnership Award, a Tertiary Education Scholarship, and the Anthony Rizzo Memorial ALS Research Fund facilitated by the Research Trust (RIDT) of the University of Malta.

## Conflict of interest

The authors declare that the research was conducted in the absence of any commercial or financial relationships that could be construed as a potential conflict of interest.

## Publisher’s note

All claims expressed in this article are solely those of the authors and do not necessarily represent those of their affiliated organizations, or those of the publisher, the editors and the reviewers. Any product that may be evaluated in this article, or claim that may be made by its manufacturer, is not guaranteed or endorsed by the publisher.
