# Supplementary material for: Reduced levels of ALS gene DCTN1 induce motor defects in Drosophila
Source: Front Neurosci. 2023 Jun 9;17:1164251. doi: 10.3389/fnins.2023.1164251 (PMC10289029; doi:10.3389/fnins.2023.1164251)
Supplement: Supplementary file 2 [file Data_Sheet_1.PDF]

SUPPLEMENTARY MATERIAL

Reduced levels of ALS gene *DCTN1* induce motor defects in *Drosophila*

Rebecca Borg; Paul Herrera; Angie Purkiss; Rebecca Cacciottolo; Ruben J. Cauchi*

* Corresponding Author
E-mail: ruben.cauchi@um.edu.mt

Supplementary Figure 1.
Conservation of Dctn1 and Dred proteins.

Supplementary Figure 2.
Survival of adult flies with muscle-specific knockdown of *Dred* or *Dctn1*.

Supplementary Figure 3.
Muscle- or neuron-specific knockdown of *Dred* or *Dctn1* does not disrupt larval mobility.

Supplementary Figure 4.
Genes that are alternatively spliced in response to *Dctn1* silencing.

Dataset S1 (separate Excel file).
DEGs identified by RNA-seq on *Dctn1* knockdown.

Dataset S2 (separate Excel file).
DSGs identified by RNA-seq on *Dctn1* knockdown.

Dataset S3 (separate Excel file).
DSGs with a synaptic function downstream of *Dcnt1* knockdown.

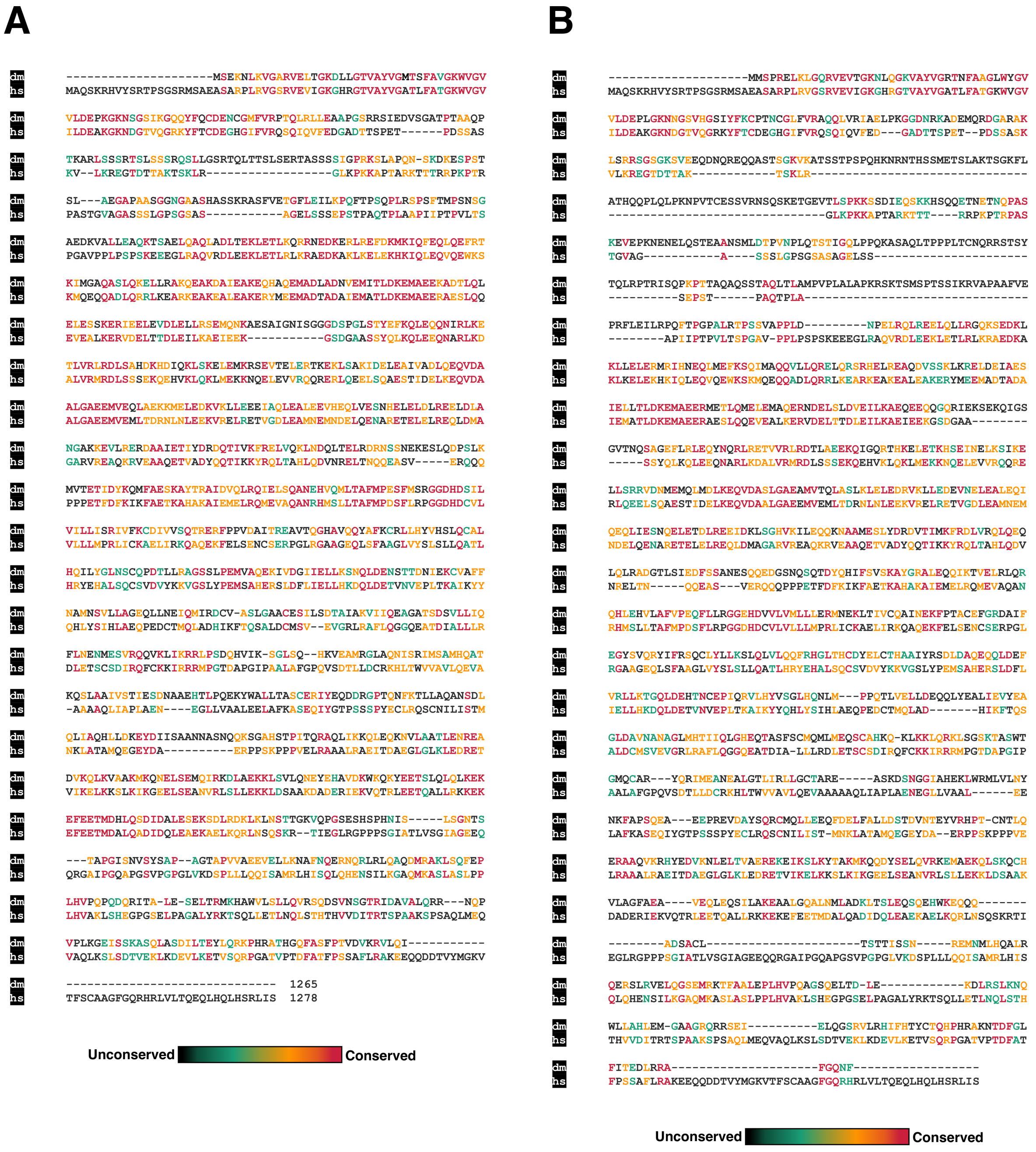

Supplementary Figure 1. Conservation of Dctn1 and Dred proteins. Protein alignment of human (hs) DCTN1 and *Drosophila* (ds) orthologues Dctn1 (**A**) or Dred (**B**).

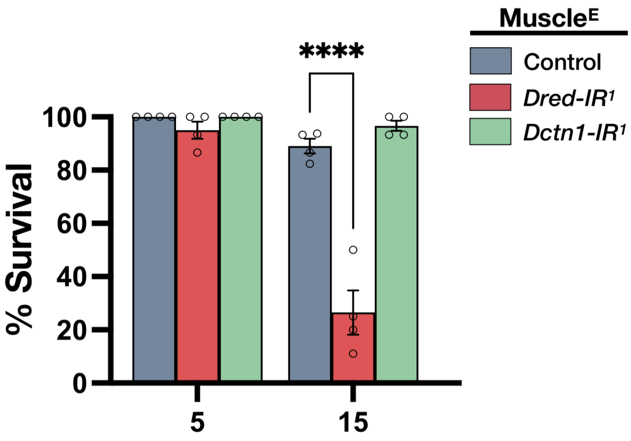

Supplementary Figure 2. Survival of adult flies with muscle-specific knockdown of *Dred* or *Dctn1*. Flies with loss of *Dred* function in muscles have reduced survival as early as day 15 post-eclosion. Each bar represents the mean ± SEM of 4 independent experiments superimposed on the bars (for each genotype, *n* ≥15 flies/replicate). Significance was tested by two-way ANOVA with Dunnett's *post hoc* test (*****p*<0.0001). Enhanced knockdown was achieved by co-expression of *Dcr-2* (indicated by E in superscript). Control expressed both driver (*Mef2*-GAL4) and *Dcr-2*.

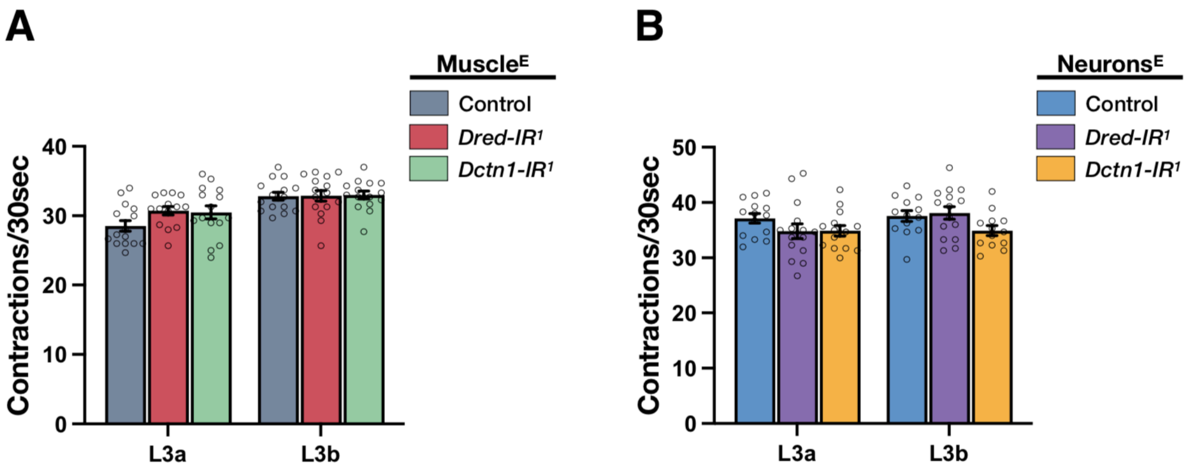

Supplementary Figure 3. Muscle- or neuron-specific knockdown of *Dred* or *Dctn1* does not disrupt larval mobility. (**A**) Body wall contraction rate of L3 larvae with muscle-selective downregulation of *Dred* or *Dctn1* assessed at 72 hours (L3a) and, subsequently, 96 hours (L3b) after egg laying (*n* ≥13/genotype). (**B**) Body wall contraction rate of L3 larvae with neuron-selective downregulation of *Dred* or *Dctn1* assessed at 72 hours (L3a) and, subsequently, 96 hours (L3b) after egg laying (*n* ≥13/genotype). For both (**A**) and (**B**), each bar represents the mean ± SEM of several independent experiments superimposed on the bars.

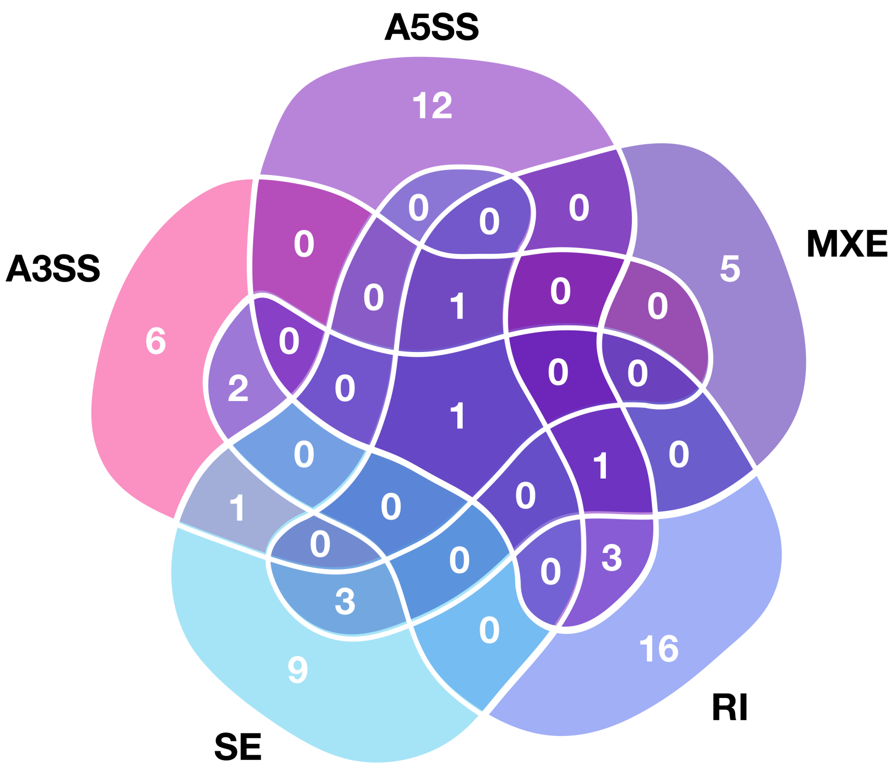

Supplementary Figure 4. Genes that are alternatively spliced in response to *Dctn1* silencing. Venn diagram showing overlap of differentially spliced genes (DSGs) across the five modes of alternative splicing including alternative 3’ splice site (A3SS), alternative 5’ splice site (A5SS), mutually exclusive exon (MXE), retained intron (RI) and skipped exon (SE).
